# External-Stimuli-Assisted Control over Assemblies of Plasmonic Metals

**DOI:** 10.3390/ma11050794

**Published:** 2018-05-15

**Authors:** Kanako Watanabe, Kotaro Kuroda, Daisuke Nagao

**Affiliations:** Department of Chemical Engineering, Tohoku University, Sendai 980-8579, Japan; kanako.w@dc.tohoku.ac.jp (K.W.); kotaro.kuroda.t4@dc.tohoku.ac.jp (K.K.)

**Keywords:** external stimuli, plasmonic nanoparticles, localized surface plasmon resonance, surface-enhanced Raman scattering, assembled state, magnetic field, electric field

## Abstract

Assembly of plasmonic nanoparticles (NPs) in suspensions is a promising approach for the control of optical and sensing properties that depend on the assembled states of plasmonic NPs. This review focuses on the controlling methods to assemble the NP via external stimuli such as pH, temperature, light, magnetic field, and electric field. External stimuli are introduced as powerful tools to assemble the NPs because of various operational factors, such as the intensity, application time, and frequency, which can be employed. In addition to a summary of recent studies on the controlling methods, a future study on the reversible control over assembled states of the plasmonic NPs via external stimuli is proposed.

## 1. Introduction

**Figure 1:**
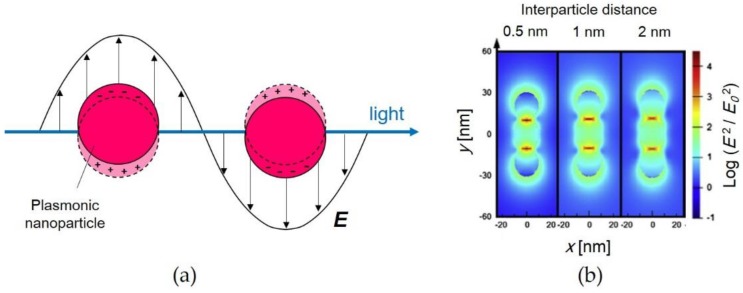
(**a**) Scheme of localized surface plasmon resonance (LSPR) of plasmonic nanoparticles (NPs); and (**b**) mappings of electric field intensities between gold (Au) NPs for different interparticle distances: 0.5, 1, and 2 nm. Reproduced with permission from Reference [16] (Copyright 2014 Elsevier).

Localized surface plasmon resonance (LSPR) is a remarkable characteristic of plasmonic nanometals such as silver and gold [1,2]. The electric field on the surface of metal nanoparticles (NPs), which is caused by the formation of dipoles in the NPs, is induced via light irradiation (see Figure 1a). Light with a specific wavelength is absorbed when the wavelength of the electron oscillation resonates with that of the incident light. The LSPR wavelength depends on the size [3] and shape [4,5] of plasmonic NPs. For instance, the LSPR peaks from spherical gold nanoparticles (Au NPs) are red-shifted by increasing the NP size [6]. Since gold nanorods exhibit dual LSPR peaks corresponding to long and short axes, the optical properties of the nanorods depend upon the aspect ratio [7]. Gold nanostars [8,9] and nanoplates [10,11] have been studied in recent years because of their shape-dependent colors. The intensity of the electric field induced on a NP surface is dramatically enhanced by bringing NP surfaces close together to form interstices called “hotspots” [12,13]. The energy level of the free electrons of the NPs at the hotspots with the enhanced electric field is lower than that of a single NP dispersed in solution [14,15]. According to the mappings of the electric field intensities shown in Figure 1b, the intensities depend upon the distance between the NPs [16].

Surface-enhanced Raman scattering (SERS), which is a phenomenon of plasmonic NPs, has received enormous interest in biomedical fields since its discovery in the 1970s [17,18]. Different mechanisms that involve electromagnetic enhancement and/or chemical enhancement on metal surfaces have been proposed thus far to explain the SERS phenomenon [19,20]. Because Raman intensities of specific molecules on NP surfaces are prominently enhanced, SERS is a powerful tool for developing highly sensitive sensors, particularly for biomolecules [21,22,23]. Previous reports indicated that the hotspots formed by assembled plasmonic NPs play an important role in SERS [24,25]. The Raman intensities at the hotspots were much higher than those on the surface of single plasmonic NPs.

Assembly of plasmonic metals is an effective way to create hotspots. A combination of top-down and bottom-up lithographic approaches has been commonly employed to provide a precise pattern of the metals on a substrate [26,27]. Electron beam lithography is one of the most effective top-down approaches to fabricate periodic structures of plasmonic metals [28,29]. Template-assisted self-assembly of the NPs has been reported as the bottom-down approaches [30,31,32]. Assembly techniques for the NPs driven by the capillary force at fluid interfaces have also been proposed in recent years [33,34]. The formation of chemical bonds between clustered plasmonic NPs is one of the bottom-up approaches for creating hotspots [35,36]. A new approach to fabricate the assemblies of the NPs using external stimuli, which are defined as switchable external energy sources used to control the assembly of NPs, have been reported [37]. In this review, previous reports on assembling plasmonic NPs are classified according to their different external stimuli: pH, light, temperature, magnetic field, and electric field.

## 2. Control over the Assembly of Plasmonic NPs via External Stimuli

Controlling the assembly of plasmonic NPs via external stimuli offers several advantages, including high operability over the states of the NP assembly and reversible control over the assembled states. In addition to circumstantial stimuli, such as pH of the solution and temperature, manually and locally switchable stimuli, such as light, electric field, and magnetic field, are used for controlling NP assembly.

### 2.1. pH

**Figure 2:**
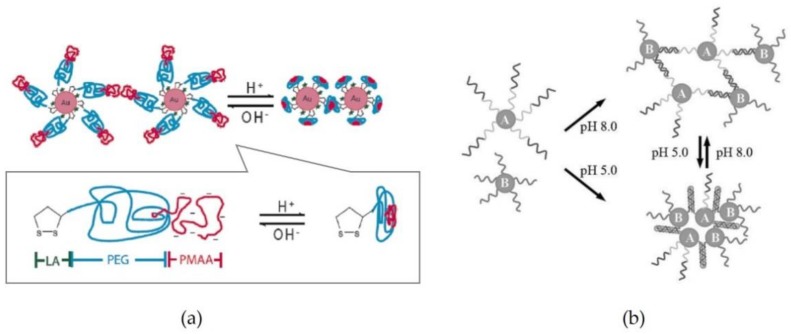
Schemes of pH-assisted assembly system of Au NPs functionalized with PMMA segments (**a**) and DNAs (**b**). Reproduced with permission from Reference [40] (Copyright 2009 American Chemical Society) (**a**) and Reference [44] (Copyright 2008 John Wiley & Sons) (**b**).

The dispersion and aggregation of NPs are affected considerably by suspension conditions such as the particle concentration and temperature. For NPs surface modified with stabilizers that are protonated and/or deprotonated in a solution, the suspension pH is an important factor to control the assembled state of the NPs. Glutathione (GSH) [38,39], polymethacrylic acid (PMAA) [40], and poly(acrylic acid) (PAA) [41,42,43] have been used as surface modifiers for pH-responsive assembly of NPs. Qian et al. [40] reported a pH-assisted system for the assembly of Au NPs modified with PMAA segments (see Figure 2a). The PMAA segments are hydrophilic above pH 4 due to the ionization of the carboxylic acid groups, and the segments on the particle surfaces expand in solution. In contrast, at pH 3 or lower, the segments condense due to increased hydrophobicity. By transitioning between an expanded and a condensed state, the assembly of the Au NPs modified with PMAA was successfully controlled via pH adjustment.

The formation/dissociation of entangled springs of DNA are controlled by adjusting the pH values. Chen et al. [44] prepared Au NPs functionalized with two types of 5′-thiolated oligonucleotides (DNA A and DNA B), which are described as NP-A and NP-B in Figure 2b. Because the formation/dissociation of the DNA springs were changed by switching the solution pH between 8 and 5, the assembled state of the Au NPs was obtained at pH 5. The Au NPs could be reassembled at pH 5 due to the reversible formation/dissociation in the solution pH range of 5 to 8, although the solution pH had to be preciously adjusted.

Since pH values vary throughout the body depending on the organs and cell type, pH-controllable assemblies of plasmonic NPs have a potential for biomedical applications such as biosensing, bioimaging, and thermotherapy. For example, cancer cells are well known to exhibit a lower pH than normal cells [45]. Nam et al. [46] proposed a new biomedical material using Au NPs with a pH-responsive ligand. In this study, 10-nm Au NPs successfully modified with the ligand and doxorubicin (DOX) were prepared for a drug delivery system. The modified NPs with DOX exhibited a net negative surface charge in a blood environment (pH 7.4) because of a carboxylic terminal of the ligand. The modified NPs that penetrated into cancer cells at pHs lower than 5.5 could release DOX from their ligands. Following the DOX release, Au NPs were clustered in cancer cells because of surface charge neutralization caused by the hydrolysis of the ligand. It was also demonstrated that the clustered Au NPs can be applied to photothermal therapy. These experimental results proved the use of the functionalized Au NPs for both drug delivery and cancer therapy applications. NPs modified with proteins, which are called nanoparticle-protein coronas, have the potential to affect the assembled states of Au NPs in biological systems [47]. However, according to a previous report, the negatively charged ligands with carboxylic terminals could prevent the aggregation of the protein coronas in blood vessels [48].

### 2.2. Temperature

**Figure 3:**
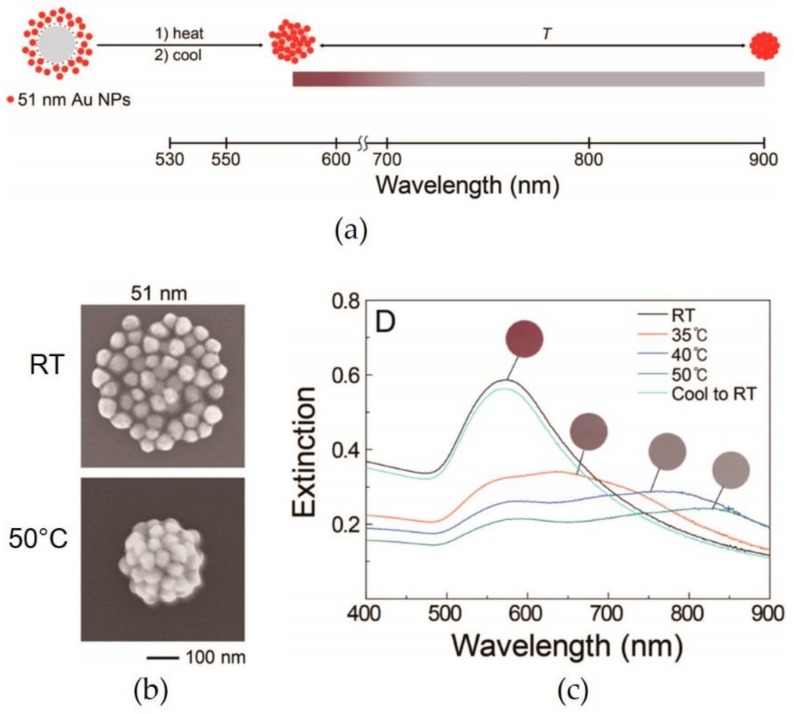
(**a**) Scheme of the temperature-assisted control over the Au NP assembly supported in a PNIPAM gel; (**b**) SEM images of the poly(*N*-isopropylacrylamide) (PNIPAM) gels observed at room temperature (RT) and at 50 °C; and (**c**) UV–Vis spectra of the gels measured at various temperatures. Reproduced with permission from Reference [52] (Copyright 2014 American Chemical Society).

Heating and cooling are circumstantial stimuli that are commonly applied to temperature-responsive materials. For instance, poly(*N*-isopropylacrylamide) (PNIPAM) is well known as a temperature-responsive polymer, having a lower critical solution temperature (LCST) of 32 °C at which PNIPAM exhibits a volume-phase transition [49,50,51]. Figure 3a shows the PNIPAM gels supporting Au NPs reported by Lim et al. [52]. The size of the gels, incorporating Au NPs, decreased after heating at 50 °C, suggesting that the distance between the NPs was closer than that obtained at room temperature (see Figure 3b). According to the UV–Vis spectra measured at various temperatures in Figure 3c, the optical properties of the gels were thermally controllable. In addition, the results indicated that the assembled states of the gels were reversibly controlled by heating and cooling.

**Figure 4:**
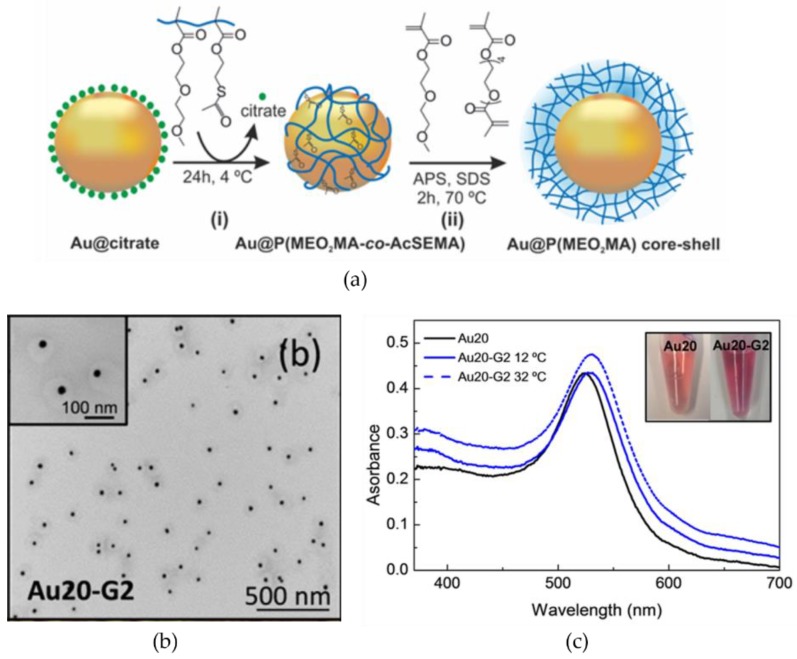
(**a**) Scheme of the preparation for Au@P(MEO_2_MA) particles; (**b**) TEM image of the particles; and (**c**) the optical property of the particles measured at different temperatures. Reproduced with permission from Reference [54] (Copyright 2016 American Chemical Society).

The polymers and copolymers composed of oligo(ethylene glycol) methacrylate (OEGMA) have recently been highlighted as promising thermosensitive polymers [53]. The OEGMA has several advantages, including good biocompatibility and tunable LCST which can be modulated by copolymerizing with other polymers. Guarrotxena et al. [54] demonstrated Au NPs coated with poly(2-(2-methoxyethoxy)ethyl methacrylate) (P(MEO_2_MA)) and Au@P(MEO_2_MA) particles, as shown in Figure 4a,b. The particles were shrunken at a temperature above their LCST (approximately 22 °C). As shown in Figure 4c, the optical properties of the particles in their suspension were modulated by temperature, suggesting that the circumstantial parameter of temperature can be used to tune the distance between NPs. Response time of thermosensitive polymers should be improved toward the application of plasmonic NPs to optical devices that are required to have quick responses. Employing thermosensitive polymers with low LCSTs has the potential to achieve quick control over the assembly of NPs between heating and cooling.

### 2.3. Light

Irradiation of light with a specific wavelength offers a variety of operational parameters, such as irradiation intensity and time. For the plasmonic NP surfaces modified with light-responsive molecules, the application of light has been used as a trigger to induce the aggregation or dispersion of plasmonic NPs [55,56,57]. Azobenzene is commonly employed as a light-responsive molecule because *cis-* and *trans-*conformations can be changed by using light irradiation [58,59]. Trans-azobenzene is isomerized into its *cis-*isomer with 365-nm light. The *cis-*isomer has a higher dipole moment than that of the *trans-*isomer, inducing the cluster formation of the NPs modified with azobenzene. The *cis-*isomer can be switched to the *trans-*isomer using 420-nm light, and its conformation is controlled by switching the irradiation wavelength.

Spiropyran, which is commercially used as a photochromic compound, is also a remarkable light-responsive molecule [60,61]. Kundu et al. [60] proposed a reversible system for controlling the assembly of Au NPs with spiropyran. Although the assemblies of Au NPs coated with spiropyran are unstable, they become stable in solution by irradiation with blue light at a 420-nm wavelength. The stability of the NPs is induced by a deprotonation and ring closure reaction of spiropyran by the light. This study also showed that the assembled state of the NPs was restored to the dispersed state of the NPs via dark incubation.

**Figure 5:**
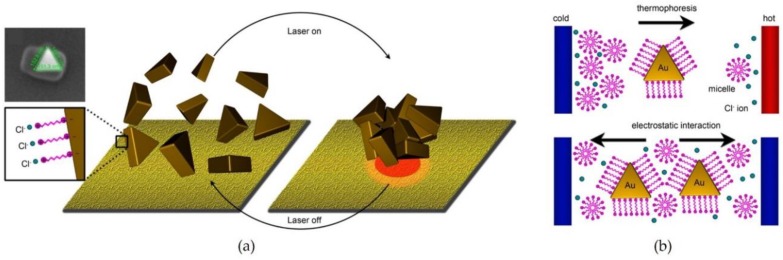
(**a**) Schemes of the laser-induced assembly system of gold nanoplates modified with CTAC; and (**b**) the movement of the nanoplates induced by thermophoresis on the substrate. Reproduced with permission from Reference [62] (Copyright 2016 American Chemical Society).

Since it is possible that the photoresponsive molecules are damaged by high-powered light, an alternative approach without using the molecules was developed to control the assembled states of plasmonic NPs. Lin et al. [62] proposed an assembly system of Au nanoplates with a laser low enough (power: 0.1 mW) not to damage non-photoresponsive molecules, such as cetyltrimethylammonium chloride (CTAC). Irradiation of plasmonic substrate with a 532-nm laser locally heated the substrate and induced thermophoresis of the Au nanoplates on the substrate, as shown in Figure 5. In this system, the assembled states of the NPs could be controlled reversibly because the temperature difference is gradually homogenized after turning the laser off.

**Figure 6:**
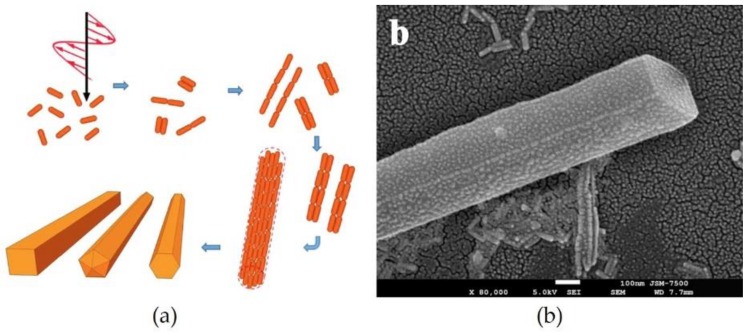
(**a**) Scheme of the formation of the Au nanowires; (**b**) and SEM image of the nanowire obtained by light application. Reproduced with permission from Reference [63] (Copyright 2017 Springer Nature).

Yu et al. [63] successfully fabricated Au nanowires comprising nano-sized Au rods using near-infrared light (see Figure 6). The nanorods were regularly aligned with their ends close together, and the aligned assemblies were gathered by irradiation with 785-nm wavelength light. The advantage of this system is that neither irradiation with strong light nor addition of chemicals are required for the formation of assemblies. In that report, the determination of the mechanism of the nanowire formation was the scope of their future study.

### 2.4. Magnetic Field

Magnetic field can be used as an external stimulus to locally induce the aggregation and dispersion of NPs in suspension. Functionalization of plasmonic NPs with magnetism has been studied as a means to control the assembled states of plasmonic NPs by an external magnetic field. Magnetic-plasmon particles, which have both magnetic and plasmonic properties, have been previously reported [64,65]. The particles are expected to be applied to biomedical fields, such as magnetic resonance imaging, photothermal therapy, and drug delivery systems.

One-dimensional chain structures of magnetically-responsive particles are formed by attractive forces, namely magnetic dipole-dipole force and van der Waals force, under an external magnetic field. Because the strong attractive forces induce the formation of clusters of the NPs under a magnetic field, repulsive forces to redisperse the particles are required to achieve reversible control over the assembly of the particles. Xue et al. [66] calculated the interaction forces between magnetite NPs coated with Au shells (Fe_3_O_4_@Au) via Monte–Carlo simulations. According to this study, the interaction energy ($U_{int}$) under a magnetic field is represented by Equation (1) as follows:

(1) $$U_{int}=U_{dd}+U_{e}+U_{vdw}$$

where $U_{dd}$ is the magnetic dipole-dipole energy, $U_{e}$ is the electrostatic repulsion energy, and $U_{vdw}$ is the van der Waals potential energy. After the magnetic field is removed, the Fe_3_O_4_@Au particles redisperse in their suspension because the $U_{e}$ becomes dominant.

**Figure 7:**
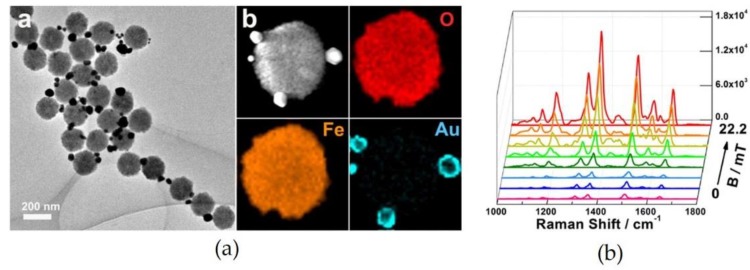
(**a**) TEM image and High-angle Annular Dark Field (HAADF)-STEM image of the Fe_3_O_4_@Au particles; and (**b**) Raman spectra of probe molecules (R6G) measured under the application of a magnetic field. Reproduced with permission from Reference [67] (Copyright 2014 Springer Nature).

Several groups experimentally demonstrated that the assembly of Fe_3_O_4_@Au can be controlled by switching the magnetic field ON/OFF states. Hu et al. [67] reported the Raman signals of probe molecules on the Fe_3_O_4_@Au particle surface were enhanced by increasing the intensity of a magnetic field (see Figure 7b). This result suggested that the hotspots for SERS should be formed at the interstices between the particles under the field application. Furthermore, they indicated the SERS effect could be changed reversibly by switching the ON and OFF states of the field.

**Figure 8:**
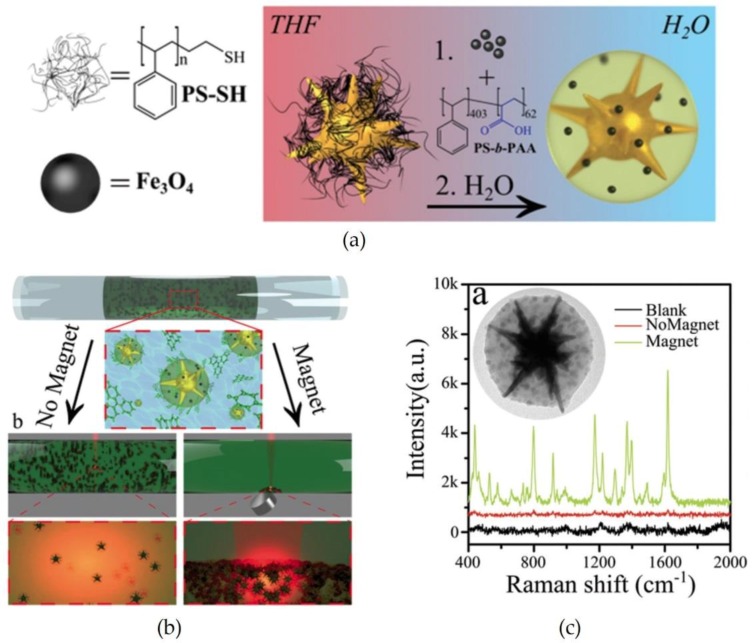
(**a**) Schemes of the preparation for Au nanostars coated with polymer shells consisting of magnetic NPs; (**b**) the assembly of the particles near a magnet; and (**c**) Raman spectra of probe molecules (Marachita Green) measured with the magnetic field. Reproduced with permission from Reference [68] (Copyright 2015 The Royal Society of Chemistry).

Porta et al. [68] synthesized Au nanostars coated with polymer shells comprising magnetic NPs as shown in Figure 8a. In the case wherein no magnetic field was applied, the particles disperse in their suspension, whereas the particles accumulate near a magnet. As shown in the Raman spectra in Figure 8c, the targeted peaks of probe molecules were dramatically enhanced under the application of a magnetic field.

### 2.5. Electric Field

Since metal NPs exhibit high responsivity to an external electric field compared with other NPs, the application of the field is a promising approach toward the control of the assembly of plasmonic NPs [69,70]. In addition, the conditions of an electric AC field (e.g., field strength, frequency, and application time) are easily adjusted. The dielectric force is induced by the application of the field, causing the movement of the NPs between electrodes in solution. The time average of the dielectric force is represented by Equation (2) as follows [71,72]:

(2) $$\langle {\vec{F}}_{DEP}\left(t\right)\rangle =2\pi \epsilon_{m}a^{3}Re\left[K\left(\omega \right)\right]\nabla {\left|{\vec{E}}_{rms}\right|}^{2}$$

where $\epsilon_{m}$ is the permittivity of the medium, a is the radius of the nanoparticle, and ${\vec{E}}_{rms}$ is the rms value of the electric field. K(ω) in Equation (2) is the Clausius–Mossotti factor represented by Equation (3) as follows [71]:

(3) $$K\left(\omega \right)=\frac{\epsilon_{p}-\epsilon_{m}-\frac{j}{\omega }\left(\sigma_{p}-\sigma_{m}\right)}{\epsilon_{p}+2\epsilon_{m}-\frac{j}{\omega }\left(\sigma_{p}+2\sigma_{m}\right)}$$

where $\epsilon_{p}$ is the permittivity of the nanoparticle,$\;j^{2}$ is −1, ω = 2π*f*, and f is frequency. $\sigma_{p}$ and $\sigma_{m}$ are the conductivities of the nanoparticle and the medium, respectively.

At low frequencies, AC electroosmosis is produced by fluid flow between the electrodes. Fluid velocity near the electrodes is represented by Equation (4) as follow [73]:

(4) $$\langle v\rangle =\frac{1}{8}\frac{\epsilon_{m}V_{0}^{2}\Omega^{2}}{\eta x{\left(1+\Omega^{2}\right)}^{2}}$$

where $V_{0}$ is the potential applied to the electrode, η is the solution viscosity, x is the coordinate axis along the electrode surface with its origin at the center of the electrode gap, and the dimensionless frequency Ω is represented by Equation (5) as follow:

(5) $$\Omega =\omega x\frac{\varepsilon }{\sigma }\frac{\pi }{2}d$$

where d is the Debye–Hückel length represented by Equation (6) as follows [62,74]:

(6) $$d={\left(\frac{\epsilon_{m}kT}{2Ie^{2}}\right)}^{1/2}$$

where k is Boltzmann’s constant, T is temperature, e is the protonic charge, and I is the ionic strength.

**Figure 9:**
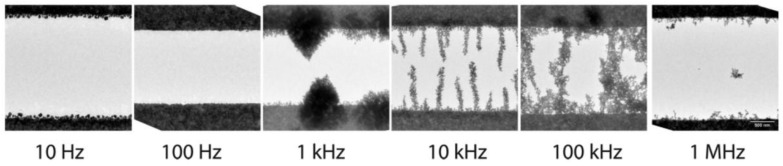
TEM images of Au NP assemblies formed by the application of an AC electric field at various frequencies: 10 Hz–1 MHz. Reproduced with permission from Reference [71] (Copyright 2008 American Chemical Society).

Gierhart et al. [71] investigated frequency-dependence on the assembled states of Au NPs via the application of an electric field with various conditions. The frequencies ranging from 10 Hz to 1 MHz were applied to Au NPs in their suspensions. As shown in Figure 9, the assemblies of the NPs were varied according to the applied frequencies. Although additional experimental setups, including electrodes and a function generator, are required to generate the external AC electric field, the high operability of the field strength and the frequency can be advantageous to vary the assembly of the NPs.

Vutukuri et al. [75] successfully controlled the assembly of gold nanoplates by the application of an electric field. The ordered assemblies of the nanoplates were fabricated by gathering the side-to-side clusters of nanoplates that were formed by adding salt to their suspension. Since the dielectric force also depends on the size of particles (see Equation (2)), the clusters were easily assembled under the field application.

**Figure 10:**
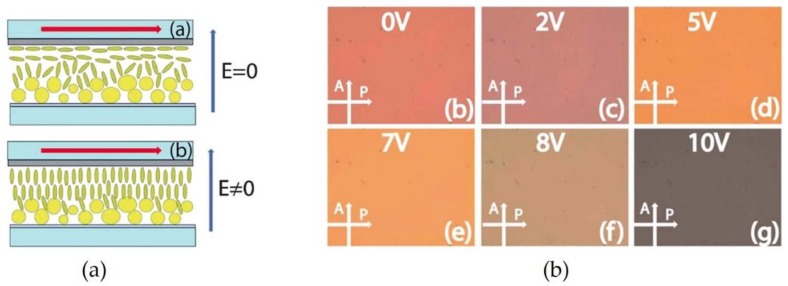
(**a**) Schemes for controlling the orientation of NLCs between glass/ITO substrates by an electric field; and (**b**) color of the substrates obtained by the field application at various field strengths: 0 to 10 V. Reproduced with permission from Reference [76] (Copyright 2012 The Royal Society of Chemistry).

Both direct control over the NP assemblies in suspension via dielectrophoresis, and indirect control over NP assemblies in nematic liquid crystals (NLCs) have been reported. Sio et al. [76] investigated an optical device comprising NLCs and Au NP arrays immobilized on a glass/Indium Tin Oxide (ITO) substrate. As shown in Figure 10a, the NLCs were regularly oriented to the direction of the applied electric field, changing the effective birefringence of the device. Due to the change of the birefringence, the colors of the substrates gradually changed while increasing the applied voltage of an electric field from 0 to 10 V.

**Figure 11:**
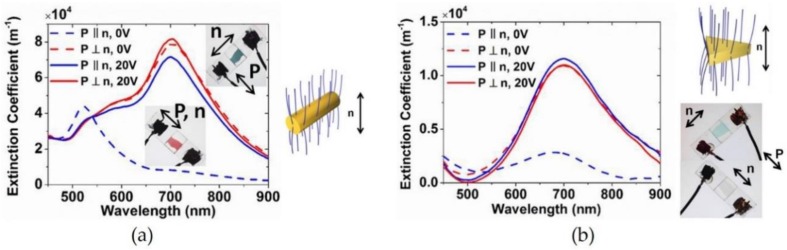
(**a**) Extinction coefficient and color changes of Au nanorods and (**b**) Au nanoplates in liquid crystals (LCs) measured with light at different polarization directions. Reproduced with permission from Reference [77] (Copyright 2015 American Chemical Society).

In recent years, the application of an electric field to control the orientation of anisotropic NPs, such as nanorods and nanoplates, has received considerable attention. Zhang et al. [77] prepared Au nanorods and Au nanoplates, which were functionalized with an orientation molecule: *N*,*N*-dimethyl-*N*-octadecyl-3-aminopropyltrimethoxysilyl chloride (DMOAP). In addition, they controlled the alignment of Au nanorods modified with DMOAP by controlling the orientation of NLCs with a field strength of 20 V. In the case of P ‖ n (P is the direction to a polarization of NPs), a 500-nm absorption peak was measured without the field application (see Figure 11a). The peak was red-shifted by the electric field with a field strength of 20 V, revealing that the alignment of the rods was caused by the orientation of LNCs in solution. As with the nanoplates in Figure 11b, the optical properties measured with and without an electric field were changed depending on the direction of the electric field component of the irradiation light. This study suggested that applying an electric field is an attractive method for controlling the alignment of anisotropic NPs indirectly using NLCs.

## 3. Summary and Outlook

In this review, we focused on the control over plasmonic NP assembly via external stimuli: pH, temperature, light, magnetic field, and electric field. The NPs used in the assembly can be classified into two groups. The first comprises plasmonic NP surfaces modified with stimuli-responsive molecules, which are controlled by external stimuli such as pH, temperature, and light. Selection of the stimuli-responsive molecules is essential to allow the NPs to be assembled by the external stimulus. The second is the plasmonic NPs without stimuli-responsive molecules, which are controlled using thermal gradients, magnetic dipole-dipole forces, and dielectrophoresis. Because a specific surface modification is not required, the latter will be able to develop facile methods for controlling the assembled states of plasmonic NPs.

Compared with conventional methods (e.g., photolithography and special agents for chemical bonding), the external stimulation of NPs is cost-effective and offers a variety of operational factors to control the assembled states of NPs. Solution-based circumstances, including solution pH and temperature, as well as application field parameters such as field strength, application frequency, and time, can be used as potential parameters to diversify the assembled states of NPs. There was no need for special experimental setups in the controls over assembled states of the NPs to facilitate the application of the methods summarized in this review.

Another advantage of employing the external stimuli is reversible control over assembly of NPs in their suspension. In addition to the attractive interactions between NPs to be assembled, repulsive interactions between NPs are required to be redispersed in suspension. To stabilize NPs electrostatically, high potentials on NP surfaces have to be maintained to overcome the NP aggregation caused by strong van der Waals forces between NPs. In the previous reports introduced in this review, the assembled states of composite particles comprised of plasmonic NPs and particles stabilized electrostatically were controlled by external magnetic fields. Almost no study on controls over plasmonic NPs with the assistance of external electric field has been reported thus far. Because of the high responsivity of the NPs to an electric field, the reversible control over the assembly of NPs by the external electric field is an urgent issue. For the realization of novel NP assemblies with various structures reversibly switched by the electric field, some physical barriers surrounding the NPs, such as thin silica shells, will be required to prevent the NP aggregation caused by strong attractive forces between NPs under application of an external electric field.
